# Suitability of Pedigree Information and Genomic Methods for Analyzing Inbreeding of Polish Cold-Blooded Horses Covered by Conservation Programs

**DOI:** 10.3390/genes12030429

**Published:** 2021-03-17

**Authors:** Grażyna Polak, Artur Gurgul, Igor Jasielczuk, Tomasz Szmatoła, Jędrzej Krupiński, Monika Bugno-Poniewierska

**Affiliations:** 1 Department of Horse Breeding, National Research Institute of Animal Production, Krakowska 1, 32-083 Balice, Poland; jedrzej.krupinski@iz.edu.pl; 2 Office of the Director for Scientific Affairs, National Research Institute of Animal Production, Krakowska 1, 32-083 Balice, Poland; 3 Center for Experimental and Innovative Medicine, University of Agriculture in Krakow, Rędzina 1c, 30-248 Kraków, Poland; artur.gurgul@urk.edu.pl (A.G.); igor.jasielczuk@urk.edu.pl (I.J.); tomasz.szmatola@urk.edu.pl (T.S.); 4 Department of Animal Reproduction, Anatomy and Genomics, University of Agriculture in Kraków, al. Mickiewicza 24/28, 30-059 Kraków, Poland; monika.bugno-poniewierska@urk.edu.pl

**Keywords:** horse, inbreeding, SNP, genomic, pedigree

## Abstract

Traditionally, pedigree-based relationship coefficients were used to manage inbreeding and control inbreeding depression that occurs within populations. The extensive incorporation of genomic data in livestock breeding creates the opportunity to develop and implement methods to manage populations at the genomic level. Consequently, the realized proportion of the genome that two individuals share can be more accurately estimated instead of using pedigree information to estimate the expected proportion of shared alleles. To make use of this improvement, in this study we evaluated the genomic inbreeding measures in the Polish conserved cold-blooded horse population and compared the data with the traditional measures of inbreeding. Additionally, an ancestry fractions/proportions from Admixture software were tested as an estimate of lineage (ancestry coefficient) used for horses qualifying for the conservation program. The highest correlation of pedigree-based (F_PED_) and genomic inbreeding estimates was found for F_ROH_ (runs of homozygosity-based F coefficient) and F_UNI_ (F coefficient based on the correlation between uniting gametes). F_ROH_ correlation with F_PED_ tended to increase as the number of generations registered as pedigree increased. While lineage and gene contributions (Q) from Admixture software correlated, they showed poor direct compliance; hence, Q-value cannot be recommended as the estimate of pedigree-based lineage. All these findings suggest that the methods of genomics should be considered as an alternative or support in the analysis of population structure in conservative breeding that can help control inbreeding in rare horse populations.

## 1. Introduction

The accuracy of inbreeding estimations based on pedigree information depends on the precision of breeding documentation. However, mistakes often appear due to imprecise records and limited knowledge of the origin. Ancestry information is difficult to obtain even for some registered animals because (1) one or more paths of their pedigrees may trace to or through foreign herd books, and (2) early ancestry information may not be recorded electronically [1]. Research has often ignored these problems and assumed that animals are not inbred and are unrelated if ancestry is unavailable [1]. Therefore, there is a need to develop a method that will allow a more accurate determination of the degree of inbreeding, and thus the risk status. This is especially important in small local populations possessing unique and valuable features.

In recent years, the results of genomic and pedigree-based studies have been compared to estimate inbreeding, especially for populations in danger of extinction. Kardos et al. [2] used computer simulations to test whether the realized proportion of the genome that is identical by descent is predicted better by the pedigree inbreeding coefficient or by genomic measures of inbreeding. Results show that marker-based measures of IBDG are substantially more precise and often less biased than FP. Ablondi et al. [3] evaluated the loss of genetic variability in the Bardigiano breed, based on linkage disequilibrium and provided the first genome-wide scan of genetic diversity and selection signatures in an Italian native horse breed, using the average inbreeding based on runs of homozygosity (ROH). Other work, completed by Mancin et al. [4], investigated the genetic diversity in the Italian Heavy Horse breed by using pedigree and genomic data. Pedigree information allowed reliable estimations of inbreeding values, resulting in medium to high correlations with genomic inbreeding. Sciavo et al. [5] analyzed the distribution of ROH in pig breeds, and found that ROH better captured inbreeding information in the analyzed breeds and could complement pedigree-based inbreeding coefficients for the management of these genetic resources.

Cold-blooded horses appeared in Poland in the second half of the 19th century. It was related to the economic exigence and associated with the import of sires from Western Europe. The main breeds included Ardennes, Belgian and Breton, while the less used breeds included North Swedish, Russian, Døle and Mur-insulan, as well as single documented cases of Fjord and Canadian horses and Boulonnais and Jutland stallions [6,7]. In the first years of the 20th century the Sokólski Center was established in the north-east region of Poland, where Breton and Norfolk–Breton stallions were allowed to mate [8]. Another center was created for Sztumski horses in East Prussia. There, cold-blooded mares were mated with German (Rhine–Belgian), French and Belgian stallions, contributing to the creation of the heaviest type of cold-blooded horses in Poland. In the first volume of the Polish stud book, edited in 1964 [9], there were 167 mares and 147 stallions of the Sokólski type, as well as 268 mares and 204 stallions of Sztumski type, which are breeds that are still present in horse pedigrees. According to Chrzanowski [10], in the 1970s there were about 600 lines established by Ardennes, Belgian and Breton stallions located in north-east Poland.

The pedigree information of these populations can be incomplete as the divisions into local breeds were removed in the second volume of the stud book [11]. All individuals were identified as Polish draft horses, which meant that local breeds of cold-blooded horses did not exist officially for almost 50 years. Currently, in the conservation programs, introduced in 2008 [12,13] the main objectives have been to maintain the genetic variability. 

In the genetic resources conservation program, only horses that have been entered in the stud book of the Polish draft horse, have an appropriate pedigree and have a conformation that conforms to the breed standard can participate. In the gene pool of Sztumski and Sokólski horses the most important contribution has come from Ardennes, Polish cold-blooded (z) and unknown (NN) horses, followed by Belgian horses. This influenced a relatively high number of founders, namely 1139 for Sztumskis and 1118 for the Sokólski population, with the effective number of founders of 156.9 and 111.4, respectively. Additionally, the data indicates that only one third (35.8% Sztumski; 38.1% Sokólskie) of the gene pool in both populations comes from local horses, and as much as two thirds from imports [14]. It is known that there was a relationship in the group of imported stallions, especially Ardennes, but it was not displayed in the database, and thus their inbreeding was considered equal to zero. This means that the volume of inbreeding could be underestimated. The analysis of the mean coefficient of inbreeding indicated 1.54 for Sztumski and 1.56 for Sokólski horses [14], with a slight upward trend, not exceeding 0.29–0.31%/year and ranging, for single animals, between 0% and 32.7%. The main group of Sztumski (71%) and Sokólski (77%) horses accounted for the lowest range of inbreeding, from ≥0 to 3.13%. Only 2% of the horses with an inbreeding coefficient exceeding 10% were found. The high level of inbreeding often resulted from the incestuous mating between parents and progeny or from the mating of half-siblings [14]. 

The generation intervals for both populations were similar—7.46 years for Sztumski and 7.24 for Sokólski horses. The pedigree completeness for five generations was respectively 98.79 and 98.24 and for all known generations, namely 16, was 51.11% and 45% [14]. From the fourth generation back they appeared as imported horses, the pedigrees of which, according to the assumptions, were not entered into the database and these foreign horses were considered as founders.

The aim of this study is to assess the concordance and mutual relationships between pedigree-based and single nucleotide polymorphism (SNP)-based inbreeding coefficients as well as between ancestry coefficients calculated using pedigree and genomic data. The results of this analysis will give a new toolset for the horse resources conservation program in Poland and will allow the assessment of the reliability and usefulness of genomic data for the maintenance of conserved horse populations.

## 2. Materials and Methods

In this study we compared and analyzed in detail the inbreeding estimates calculated based on pedigree data (F_PED_) and so called genomic inbreeding (realized inbreeding) measures expressed by four different coefficients. 

The data for the pedigree analysis came from the reference population, which consisted of 1694 Sokólski horses and 2042 Sztumski horses participating in genetic resources conservation programs in 2020 (Table 1).

The reference population is the one for which we defined the parameters, i.e., inbreeding coefficient. In this case they are the population covered by the conservation programs in 2020, according to the information contained in Table 1. The population of horses born between 1991 and 2018 is also the entire population of Sztumski and Sokólski horses participating in the protection programs. They are all simultaneously existing horses while also belonging to these populations. The pedigree data used in this study was provided by the Polish Horse Breeders Association. All the available pedigree information was entered into the Bio_konie horse database of the National Research Institute of Animal Production. The data set consists of 6531 pedigree horses born between 1991 and 2018. In total, in the database there was collected information on 30,331 horses that were (a) participating in conservation programs from 2008 to 2020 and (b) all their available ancestors. The pedigrees of 3736 horses of the reference population were traced back to the earliest recorded ancestors. It was assumed that the foreign animals (imported stallions and mares), which were found in the pedigrees, were considered as the founders, and their ancestors were not included in the database. Ancestors born in Poland of unknown origin (NN) were also considered as founders. The inbreeding coefficient was computed using a model developed by Meuwissen and Lou [15].

The study of genomic inbreeding was based on 175 cold-blooded horses, belonging to two types, namely Sokólski (*n* = 106) and Sztumski (*n* = 69), which were previously analyzed for genetic differentiation [15] and selection signatures [16]. 

Genomic inbreeding was evaluated based on Neogen Equine Community array (Illumina, San Diego, CA, USA) data, including genotypes for 65,157 SNPs with an average inter-marker distance in EquCab2.0 genome of 36.3 kb. SNP data were filtered as previously described [16]. In brief, the filters included a MAF (minor allele frequency) threshold of 5% and <20% of missing genotypes in the whole studied population. Additionally, SNPs with critical *p*-values for Hardy–Weinberg equilibrium (HWE) <1.0 × 10^−6^ in each breed separately were excluded. The final SNP panel of 52,023 markers was scattered across the horse genome (EquCab2.0; https://www.ncbi.nlm.nih.gov/assembly/GCF_000002305.2/) with an average inter-marker distance of 43.0 kb. For ROH detection, the filtered SNPs set was remapped to the EquCab3.0 (https://www.ncbi.nlm.nih.gov/assembly/GCF_002863925.1/) genome assembly using the UCSC Genome Browser and Lift Genome Annotations tool. This removed another 109 SNPs that did not map to the newer assembly. Based on these filtered data, four inbreeding measures were calculated, similarly as in our previous study [16,17]: (i) the usual variance-standardized relationship minus 1 (F_GRM_), (ii) method-of-moments F coefficient estimate (similar to F_IS_), (iii) F coefficient based on the correlation between uniting gametes (F_UNI_) [18], and (iv) runs of homozygosity (ROH)-based coefficient (F_ROH_, including ROH segments with lengths above 1 Mb) [19] using Plink v1.90b4 software [20]. The first three coefficients were calculated using Plink --ibc command, while the ROH-based coefficient was calculated by identification of ROH, covering a minimum of 30 SNPs, as in our previous study in cattle [21] and evaluation of a genome portion covered by ROH as proposed by McQuillan et al. [19]. 

Comparison among various measures of inbreeding was made using Spearman’s rank correlation (rho) coefficients following data distribution evaluation with the Shapiro–Wilk test. Statistical significance of differences in F coefficient between two analyzed horse types was assessed using an ANOVA test. Statistical testing was done using JASP software [22].

Moreover, a Q-value (ancestry fractions/proportions) from Admixture software [23], calculated as described in [15], were compared (using correlation analysis) with linage data used for horses qualifying for the conservation program. Lineage data were calculated from the proportions of the desirable ancestors, (e.g., in the Sokólski type, originating from ancestors born in a historical region and factors of the type—Breton and Ardennes horses), in relation to undesirable in the pedigree (Sztumski horses, Belgian and their derivatives, Fjords, Mur-insulan and other breeds not involved in the creation of Sokólski horses). This was done to evaluate whether Q-value can be used instead of lineage data in the conservation program. Similarly, for the Sztumski type, the percentage of the pedigree was estimated as the proportion of ancestors born in the historical region and the factors of the type (Ardennes, Belgian horses and their derivatives) to the undesirable ancestors, Breton, Sokólski horses, Fjords, Mur-insulins and other breeds not involved in the creation of Sztumski horses.

## 3. Results

### 3.1. Pedigree Data Analysis

**Figure 1:**
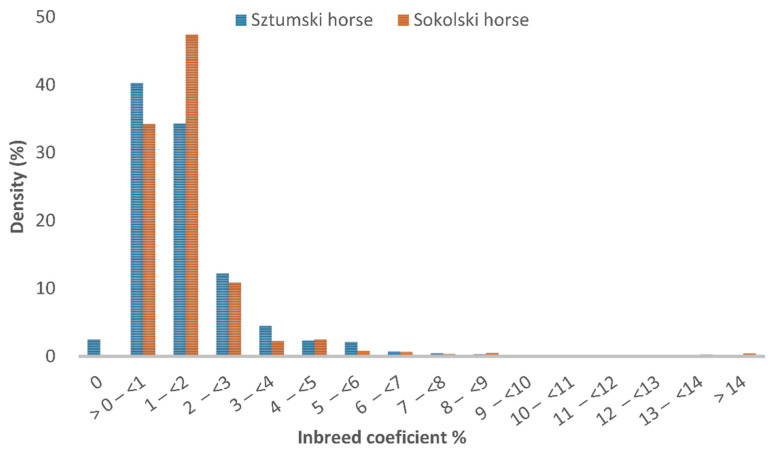
Distribution of the inbreeding coefficient in the reference population of the Sztumski and Sokólski cold-blooded horses in 2020.

**Figure 2:**
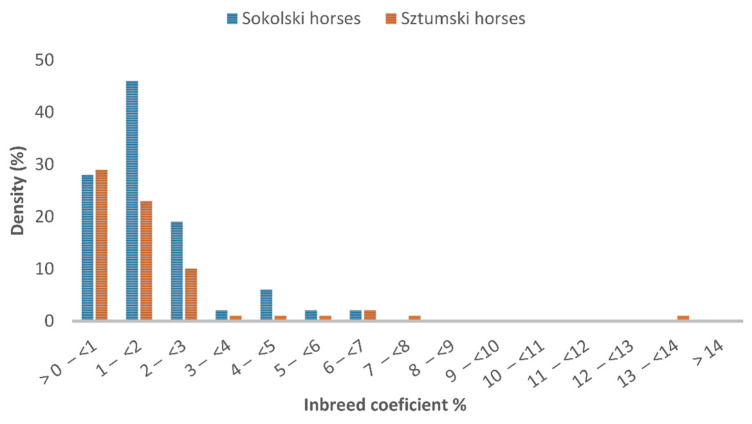
Inbreeding coefficient in 175 analyzed Sztumski and Sokólski cold-blooded horses, based on pedigree information.

The distribution of the inbreeding coefficient in the reference population of the Sztumski and Sokólski cold-blooded horses, estimated on the basis of the pedigree information (F_PED_), is presented in Figure 1 and in the analyzed population in Figure 2. 

The mean inbreeding coefficient was 0.0159 for the Sokólski population and 0.0155 for the Sztumski population. 

The inbreeding distribution of the sample from the population (175 horses were included in the genomic analysis) is very similar to the distribution in the entire population of Sztumski and Sokólski cold-blooded horses (Figure 1). Nevertheless, in the range of values from zero to <0.0313% the proportions are reversed—there is a greater share of Sztumski individuals and there are no non-inbred individuals.

The pedigree completeness index per each generation show that in the first two generations, 100% of the ancestors are present. In the third and next generation there is an increasing loss of ancestors, most often due to the fact that they are individuals of foreign breeds whose pedigrees, with the assumptions, are not entered into the database.

**Figure 3:**
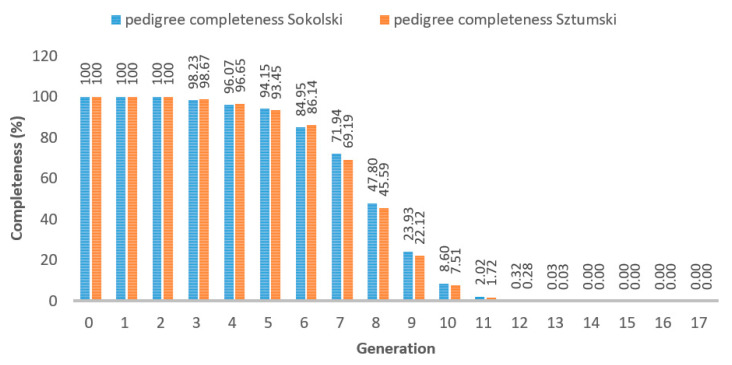
Pedigree completeness (%) in analyzed Sztumski and Sokólski cold-blooded horses, based on pedigree information.

**Figure 4:**
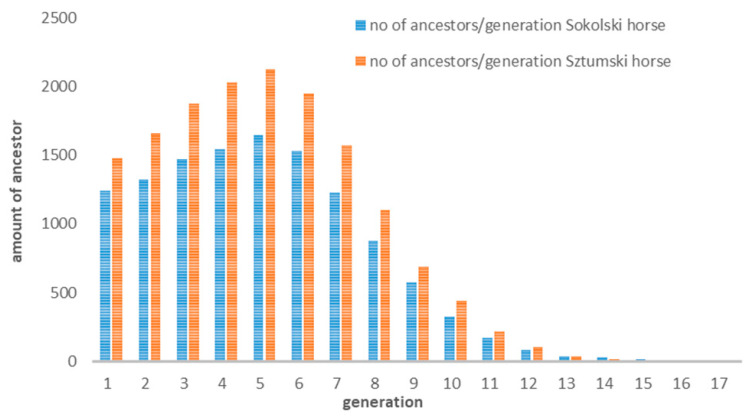
The numbers of available pedigree information in subsequent generations.

The number of available ancestors’ data in our pedigree information is presented in Figure 3 and the increase of inbreeding in subsequent generations depending on the availability of pedigree information is given in Figure 4.

**Figure 5:**
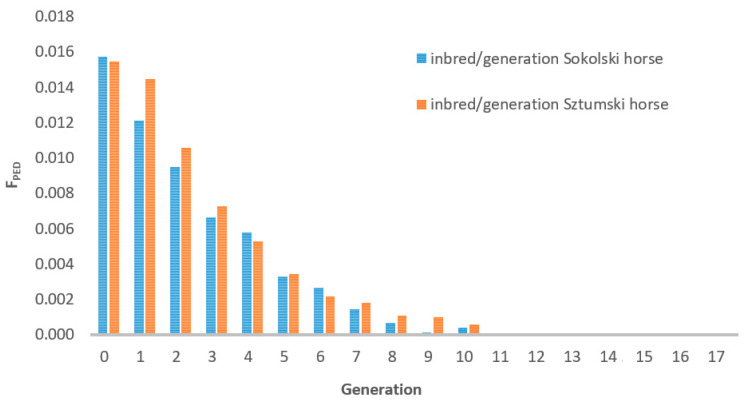
Increase of inbreeding in subsequent generations depending on the availability of pedigree information.

The visible decrease in the number of pedigree information from the sixth generation results from the appearance of numerous imported ancestors–founders, i.e., whose data was not entered into the database (Figure 4). It is also indicated by a decrease in the inbreeding coefficient going back to earlier generations (Figure 5).

### 3.2. Genomic Analysis and Its Comparison within Pedigree Data

The analyzed horse population included 175 animals born between 2008 and 2018 and with a high number of generations (between 11 and 15) registered in the pedigree data (Supplementary File 1, Figures S1 and S2). While analyzing inbreeding coefficients changes across birth years, we found that only F_PED_ data showed (expected in breeding populations) an increasing trend, while genomic measures rather showed a declining tendency (Supplementary File 1, Figure S3).

When compared to F_PED_, genomic measures of inbreeding either under (F_GRM_) or overestimated the population inbreeding (Table 2, Supplementary File 1—Figure S3). F_PED_ values ranged from 0.2 to 13%. This interval was similar to that observed for F_ROH_ (2–14.9%) and F_UNI_ (2–5.1%) coefficients. Negative inbreeding values (outbred) were observed exclusively for F_GRM_, suggesting the presence of some statistical artifacts.

Comparison of all F coefficients among the analyzed horse types (SOK and SZTUM) did not show any statistically significant differences (Table 3). Similarly, no significant differences were observed between males (n = 21) and females (n = 154) within both breeds.

The analysis of correlation coefficients among all studied F measures in the whole population showed the strongest relationship between F_ROH_ and F_UNI_ (rho = 0.919; *p* < 0.001) and the weakest between F_PED_ and F_GRM_ (0.351; *p* < 0.001). F_PED_ showed the strongest correlation with F_ROH_ and F_UNI_ with Spearman’s rank of 0.443 and 0.430 (*p* < 0.001), respectively (Table 4; Supplementary File 3).

**Figure 6:**
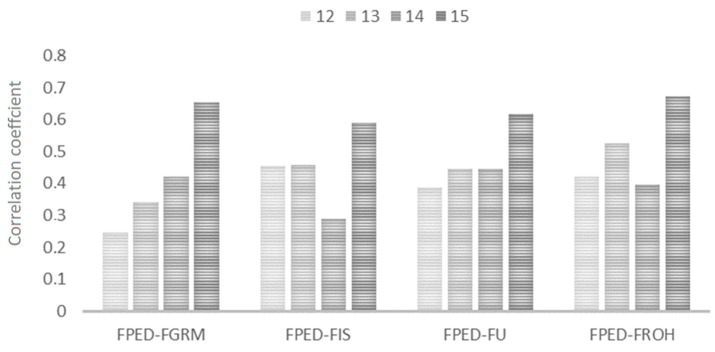
Spearman’s rank correlation coefficients among F_PED_ and genomic measures of inbreeding depending on the number of generations registered in the pedigree.

While taking into account the number of generations registered in the horse pedigrees (pedigree depth), correlations of F_PED_ with genomic data tended to increase along with the number of registered generations (Figure 6). This correlation was the highest for animals with 15 registered generations and their F_ROH_ estimates (0.673; *p* = 0.028) (Supplementary File 3).

**Figure 7:**
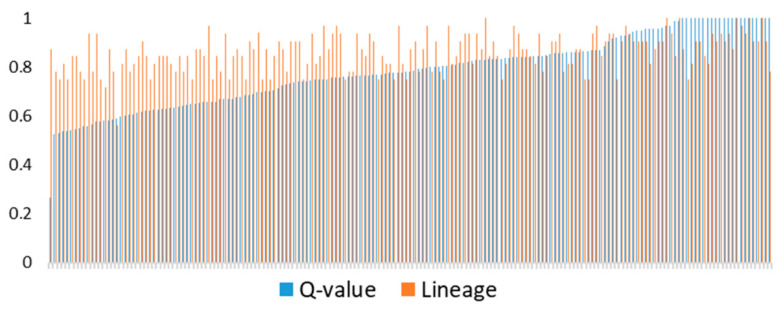
Q-value from Admixture software and lineage from pedigree data for each horse (sorted by Q-value).

Another analyzed genetic parameter was lineage (Table 5) which we correlated with Q-value (ancestry fractions/proportions) obtained from Admixture software. These data correlated with the medium value of rho = 0.385 (*p* < 0.001) (Supplementary File 4) and showed rather poor concordance for horses with outlying or extreme values (Figure 7).

## 4. Discussion

In this study we analyzed and performed various comparisons between different measures of inbreeding, including pedigree-based coefficient and genomic measures of inbreeding. It is well known that the mating of related individuals results in the inbreeding of an offspring [24]. In closed, selected populations, increase in inbreeding is inevitable and increasing inbreeding reduces genetic variation that can lead to so called “inbreeding depression” [25]. The individual inbreeding coefficient (F) is defined as the proportion of an individual’s genome that is autozygous, that has homozygous “identical by descent” (IBD) status, or equivalently the probability of a randomly sampled locus in the genome that could be autozygous. Traditionally, inbreeding coefficients are calculated from pedigree data (F_PED_) using methodology proposed by Wright [26] or more recently by the method of [27]. When pedigrees are not available or their depth or quality are poor, inbreeding coefficients can be derived from genotypic data, exploring, for example, the difference between observed and expected multilocus heterozygosity [28]. Inbreeding measures can for example be estimated by the maximum likelihood approaches [29], by methods-of-moment [30], from the diagonal elements of a genomic relationship matrix (GRM) [27], from simple heterozygosity or homozygosity measures [31], based on genotypic correlations [28] or from the proportion of the genome within ROH [19,28]. A higher level of inbreeding, that is, the proportion of genome that is IBD, brings more chance for expression of homozygous recessive deleterious alleles. These are considered to be the main cause of inbreeding depression, which reduces the fitness of animals and results in inter alia deterioration of fertility [31]. To avoid inbreeding depression, accurate and reliable estimation of inbreeding is important, especially in native and conserved populations in which a limited number of individuals are being currently used for mating or for populations that were reconstructed with a limited number of founders.

Recently, an inbreeding coefficient derived from ROH was developed and shown to be optimal for the estimation of genome-wide autozygosity and for detecting inbreeding effects [19,28,32,33]. This was confirmed in our data in which F_ROH_ showed the strongest correlation with F_PED_ data. In this analysis we used F calculated based on ROH with a length >1 Mb. This was because we wanted to capture a total animal autozygosity, including relatively distant (ancient) incidents of inbreeding which often cannot be shown in the pedigree analysis due to the lack of information. ROH are defined as contiguous homozygous regions in the genome where the two haplotypes inherited from the ancestors are identical by descent [31]. Previous studies [33], reported that the ROH segments of 2–4 Mb represent the inbreeding of distant generations of the ancestors (13–25 generations ago), which cannot usually be captured using pedigree information. The ROH segments > 8 Mb represent the proportion of autozygosity originated from ancestors that were born 6–7 generations ago and the ROH longer than 16 Mb reflect ancestors that were born 3–6 generations ago [34]. This was also visible in our data in which the correlation coefficient between F_PED_ and F_ROH_ tended to increase along with the increase of the number of generations registered in pedigree data. Similar observations were also made by other authors [33] as well as in our previous study [35]. The F_ROH_ values presented in this study are visibly lower than ones presented in our previous work [11]. This was raised as a result of SNPs re-mapping to the newest currently available horse genome assembly (EquCab3.0.). The number of ROH detected after re-mapping was clearly reduced mainly in the shortest ROH length categories, which are the most commonly identified as false positives [32,36]. However, a detailed description of this observation will be presented in our upcoming study. Similarly, moderate correlation was found in our data between F_UNI_ and F_PED_. F_UNI_ is defined as a correlation between genetic effects that gives more weight to homozygosity at rare alleles [37]. F_UNI_ is directly related to the definition of Wright [26]. It was found that in scenarios of large population sizes, such as in human populations, F_UNI_ can be an appropriate inbreeding measure to estimate inbreeding depression, whereas in scenarios of small population sizes, F_ROH_ may be more appropriate [28,38]. While F_UNI_ appears to be simpler to calculate than F_ROH_, it can be also considered as a useful measure of inbreeding in conserved horse populations. Unexpectedly low correlations were found between F_GRM_ and F_ROH_, especially when F_GRM_ belongs to the same group of methods as F_UNI_, and is based on covariances between genetic effects [28].

## 5. Conclusions

In this study, based on a conserved horse population, we showed the usefulness of genomic data to assess population inbreeding with relatively high conformance to pedigree-based estimates. We confirmed previous observations that F_ROH_ is the most similar to the pedigree-base estimates measure of inbreeding and we demonstrated its ability to reflect an ancient inbreeding. We also presented F_UNI_ as a congruent measure of inbreeding, with a similar range and values as F_PED_ and F_ROH_. While lineage and gene contributions from Admixture software correlated, it showed poor direct compliance, hence Q-value cannot be recommended as the estimate of pedigree-based lineage. All these findings suggest that the methods of genomics should be considered as an alternative or support in analysis of population structure in conservative breeding and can help in the control of inbreeding in rare horse populations.

## Figures and Tables

**Table 1:** The pedigree data of the number of Sztumski and Sokólski horses participating in genetic resources conservation programs from 2008 to 2020.

Item	Sztumski		Sokólski	
	Sire	Dam	Sire	Dam
Total horses in database	30,331			
Year of birth of oldest ancestors	1932	1938	1935	1940
Total horses under conservation (2008–2020)	6531			
	784	2594	555	2598
Reference population (under conservation in 2020)	3736			
	533	1509	335	1359

**Table 2:** Basic statistics for the analyzed inbreeding measures.

Statistics	F_PED_	F_GRM_	F_IS_	F_UNI_	F_ROH_
Mean	0.018	−0.002	0.135	0.067	0.061
Median	0.012	−0.003	0.134	0.064	0.057
Std. Deviation	0.016	0.018	0.022	0.018	0.018
Shapiro–Wilk test	0.690	0.976	0.986	0.965	0.931
*p*-value of Shapiro–Wilk	<0.001	0.004	0.072	<0.001	<0.001
Minimum	0.002	−0.044	0.069	0.023	0.026
Maximum	0.135	0.081	0.221	0.151	0.149

F_GRM_—the usual variance-standardized relationship minus 1 inbreeding coefficient; F_IS_—method-of-moments F coefficient; F_UNI_—F coefficient based on the correlation between uniting gametes, F_ROH_—runs of homozygosity (ROH)-based inbreeding coefficient.

**Table 3:** Basic statistics for the analyzed inbreeding measures with respect to the analyzed horse types.

Statistic	F_PED_		F_GRM_		F_IS_		F_UNI_		F_ROH_	
	SOK	SZTUM	SOK	SZTUM	SOK	SZTUM	SOK	SZTUM	SOK	SZTUM
Mean	0.018	0.017	−9.6 × 10^−4^	−0.004	0.137	0.133	0.068	0.064	0.063	0.058
Std. Error of Mean	0.001	0.003	0.002	0.002	0.002	0.003	0.002	0.002	0.002	0.002
Median	0.014	0.011	−0.003	−0.005	0.137	0.132	0.069	0.062	0.061	0.054
Std. Deviation	0.013	0.021	0.017	0.019	0.020	0.024	0.017	0.019	0.017	0.019
Minimum	0.002	0.002	−0.038	−0.044	0.084	0.069	0.023	0.034	0.026	0.028
Maximum	0.070	0.135	0.046	0.081	0.199	0.221	0.123	0.151	0.123	0.149

SOK—Sokólski horse; SZTUM: Sztumski horse.

**Table 4:** Correlation coefficients among all measures of inbreeding.

F Coefficient			Spearman			
			rho	*p*	Lower 95% CI	Upper 95% CI
F_PED_	-	F_GRM_	0.351	<0.001	0.213	0.474
F_PED_	-	F_IS_	0.381	<0.001	0.247	0.501
F_PED_	-	F_UNI_	0.430	<0.001	0.301	0.544
F_PED_	-	F_ROH_	0.443	<0.001	0.316	0.555
F_GRM_	-	F_IS_	0.522	<0.001	0.405	0.622
F_GRM_	-	F_UNI_	0.837	<0.001	0.787	0.877
F_GRM_	-	F_ROH_	0.733	<0.001	0.655	0.795
F_IS_	-	F_UNI_	0.888	<0.001	0.852	0.916
F_IS_	-	F_ROH_	0.875	<0.001	0.835	0.906
F_UNI_	-	F_ROH_	0.919	<0.001	0.892	0.939

CI—Confidence interval.

**Table 5:** Basic statistics for Q-value from Admixture software and linage data from pedigree.

Statistic	Q-Value to SOK Population		Q-Value to SZTUM Population		Lineage (Pedigree)	
	SOK	SZTUM	SOK	SZTUM	SOK	SZTUM
Mean	0.752	0.166	0.248	0.834	0.856	0.865
Median	0.759	0.167	0.241	0.833	0.844	0.875
Std. Deviation	0.144	0.124	0.144	0.124	0.064	0.089
Variance	0.021	0.015	0.021	0.015	0.004	0.008
Minimum	0.267	1.000 × 10^−^^5^	1.000 × 10^−^^5^	0.54	0.719	0.563
Maximum	1	0.46	0.733	1	0.969	1
Sum	79.747	11.435	26.253	57.565	90.752	59.656

## Data Availability

The data presented in this study are available on request from the corresponding author.

## Supplementary Materials

The following are available online at https://www.mdpi.com/2073-4425/12/3/429/s1, Table S1. Mean inbreeding coefficients by the year of birth in the whole population, Table S2. Spearman’s rank correlation coefficients among different measures of inbreeding depending on the number of generations registered in the pedigree, Figure S1. Number of animals by birth year in the population of genotyped animals, Figure S2. Depth of pedigrees for the population of genotyped animals, Figure S3. Changes in inbreeding coefficients (including linear trend dotted lines) across birth years of genotyped animals, Supplementary File 2. Table of correlations and correlation plots for different measures of inbreeding, Supplementary File 4. Table of correlations and correlation plots for Q-value and genetic linage.
